# Severe and Difficult Asthma: Diagnosis and Management—Challenges for a Low-Resource Environment

**DOI:** 10.1007/s12098-021-03952-w

**Published:** 2021-10-22

**Authors:** Andrew Bush

**Affiliations:** 1 Department of Pediatrics and Pediatric Respirology, National Heart and Lung Institute, Imperial College, London, UK; 2 Imperial Center for Pediatrics and Child Health, Imperial College, London, UK; 3 Department of Pediatric Respiratory Medicine, Royal Brompton Hospital, London SW3 6NP, UK

**Keywords:** Adherence, Atopy, Lung growth, Obesity, Pollution, Smoking

## Abstract

Sever﻿e and difficult asthma in a low- and middle-income country (LMIC) can relate to (a) lack of availability of basic medications; (b) potentially reversible factors such as poor adherence or comorbidities such as obesity inhibiting a good response to treatment; and (c) (rarely) true severe, therapy-resistant asthma. However, definitions of severity should encompass not merely doses of prescribed medication, but also underlying risk. The nature of asthmatic airway disease shows geographical variation, and LMIC asthma should not be assumed to be phenotypically the same as that in high-income countries (HICs). The first assessment step is to ensure another diagnosis is not being missed. Largely, political action is needed if children with asthma are to get access to basic medications. If a child is apparently not responding to low dose, simple medications, the next step is not to increase the dose but perform a detailed assessment of what factors (for example co-morbidities such as obesity, or social factors like poor adherence) are inhibiting a treatment response; in most cases, an underlying reason can be found. An assessment of risk of future severe asthma attacks, side-effects of medication and impaired lung development is also important. True severe, therapy-resistant asthma is rare and there are multiple underlying molecular pathologies. In HICs, steroid-resistant eosinophilia would be treated with omalizumab or mepolizumab, but the cost of these is prohibitive in LMICs, the biomarkers of successful therapy are likely only relevant to HICs. In LMICs, a raised blood eosinophil count may be due to parasites, so treating asthma based on the blood eosinophil count may not be appropriate in these settings.

## Introduction

Severe asthma is conventionally defined by levels of prescribed medication; either the child requires high doses to maintain control, or remains uncontrolled despite these medications. The European Respiratory Society (ERS)/American Thoracic Society (ATS) criteria [1] set the medication levels at Global Initiative for Asthma (GINA) steps 4–5. For children age 6–12 y, the threshold dose of inhaled corticosteroids (ICS) is ≥ 800 mcg/beclomethasone (DP) equivalent/d, for children aged over 12, the threshold dose is 2000 mcg. The child should also be using, or had a failed trial of, long-acting β-2 agonists (LABA), a leukotriene receptor antagonist (LTRA), and sometimes low dose (anti-inflammatory) theophylline. Uncontrolled asthma is defined as any one or more of: a) poor symptom control Asthma Control Questionnaire (ACQ) consistently ≥ 1.5, Asthma Control Test (ACT) < 20, or “not well controlled” as defined by National Asthma Education and Prevention Program (NAEPP) or GINA guidelines over 3 mo of evaluation; b) frequent severe asthma attacks, defined as ≥ 2 courses of systemic corticosteroids (≥ 3 d each) in the previous year; c) ≥ 1 serious attacks, namely least one hospitalization, intensive care unit stay or mechanical ventilation in the previous year; d) airflow limitation defined as a forced expiratory volume in 1 s (FEV_1_) < 80% predicted (in the presence of reduced FEV_1_/forced vital capacity (FVC) ratio defined as less than the lower limit of normal) following a withhold of both short- and long-acting bronchodilators. The author would also add persistent airflow limitation (PAL), which is failure to improve the above spirometry despite a course of systemic corticosteroids and acute administration of short-acting β-2 agonist (SABA). In pediatric practice, the use of standard deviation—*z* scores is preferable, with abnormal being ≥  − 1.96.

The problem with this and similar definitions is that all these numbers are arbitrary, and more importantly, the definition does not capture at least half of those with fatal or near-fatal asthma, many of whom are not on much medication but are at high risk. For example, the UK National Review of Asthma Deaths showed that underuse of ICS, overuse of SABA and failure to attend routine asthma checks were all markers of a poor outcome [2]. Hence, a definition of severe based on prescribed medication doses alone is insufficient, and must incorporate components of future risk. These encompass not merely risk of a severe asthma attack, but also risk of side-effects of medications and risks of impaired lung growth, and all-cause premature morbidity and mortality.

The WHO statement amplifies these ERS/ATS criteria [3], and is especially relevant, although also not adequately tackling future risk. It identifies three categories of severe asthma, in summary:

- Severe asthma related to inability to access basic care. Perhaps the commonest challenge for low- and middle-income countries (LMICs) is ensuring sufficient access to basic medications, especially low dose ICS (“untreated asthma”)
- Asthma which appears severe, but when basic management is optimized, the child responds well to simple medication. This is much the commonest in a high-income country (HIC). It comprises the subcategories “difficult asthma” (DA) and “asthma plus (comorbidities),” which are not mutually exclusive
- True treatment-resistant asthma. This exists, but is very rare indeed in children (“severe, therapy resistant asthma,” STRA)

Geographical context is another important consideration. No country is homogeneous, and there are islands of great wealth in LMICs, and areas of great poverty and deprivation in HICs. There are rural and urban differences, which, because farming practices differ across the world, show international differences. Asthma is not a monogenic disorder, but is the product of multiple genes and the environment, and both vary within and between countries. Hence, the nature of airway diseases may vary within and between countries. For example, in Brazil, most school age wheeze is nonatopic, with some evidence that helminth infections may be protective [4], whereas in London, pediatric helminth infections are vanishingly rare.

The *Lancet* Asthma Ccommission [5] recommended that the umbrella term ‘asthma’ should be used globally to describe a purely clinical syndrome, namely wheeze, chest tightness, and breathlessness, sometimes with excessive cough. Having identified that a child has this syndrome, the next question should be “what sort of asthma is this?” which leads to the concept of determining what are the components of the airway disease, especially what is treatable (“treatable traits”). These are as follows:

- Fixed and variable airflow obstruction: If the variability is due to bronchospasm, as opposed to other causes of variable narrowing such as airway mucus or malacia, then prescribe SABA.
- Airway inflammation: ICS are fantastic treatment, but only for eosinophilic airway inflammation, and should not otherwise be prescribed.
- Airway infection: Chronic airway infection (persistent bacterial bronchitis, PBB) should be treated with antibiotics. The child should be investigated for an underlying cause if the condition relapses or does not respond.

A difficult LMIC challenge is thus to determine the nature of the asthma from which the child is suffering, if the disease is not easily controlled [6]. This needs to be in a practical way, especially given that spirometers may not be readily available, and the blood eosinophil count, such a useful biomarker in HIC pediatric asthma, may be elevated by parasite infections in LMICs.

## Is It Asthma at All: What Is the Real Diagnosis?

One common reason for asthma apparently failing to respond to treatment at all is that the diagnosis is wrong. A thorough history and clinical examination is mandatory in all such cases [7]. Further testing depends on resources and the clinical context, but falls into two categories:

1. Tests to support an asthma diagnosis: These are to be encouraged; far too many wrong diagnoses are made, at least in HIC settings, in part because objective tests are not performed, and many asthma diagnoses missed.

- There is too often an assumption that breathlessness on exercise is due to asthma, when much the most common cause is deconditioning, whether or not the child is also obese. If in doubt, at the very least do a field exercise test and see if the child wheezes or bronchoconstricts.
- Variable airflow obstruction: Acute peak flow or spirometric response to SABA, home peak flow monitoring, exercise challenge test as above.
- Is the child atopic (absence of atopy, a pointer away from asthma): Skin prick tests, specific IgE.
- Is there evidence of eosinophilic airway inflammation: Raised blood eosinophil count (in high parasite burden areas, it may be better to use a low count as ruling out airway eosinophilia, but this has yet to be tested), elevated exhaled nitric oxide (FeNO).

These tests are also relevant to determining what sort of asthma the child has (above).

2. Test to reach an alternative diagnosis: Local LMIC knowledge is essential here, but airway compression by tuberculous lymph nodes is an important LMIC differential diagnosis, less so in HICs. Testing should be targeted and purposeful, rather than a scattergun approach of all tests on all children

## Untreated Asthma

This is possibly the biggest cause of uncontrolled asthma worldwide, but this section is relatively short as being outside this author’s expertise. The basic asthma toolkit is SABA, beclomethasone (BDP) or another cheap ICS, prednisolone, and a plastic spacer made from a drinks bottle. There is no doubt that making ICS widely available is not merely transformative of childhood respiratory health in LMICs, but also cost-effective. It is a scandal that these basic, low-cost medications are still not widely available [8]. There is likely a need for educational and other initiatives to dispel myths and eliminate the stigma of the diagnosis which exists in some parts of the world [9], in addition to the distribution of medications.

## Difficult Asthma and Asthma Plus

What is the right approach to a child with confirmed asthma who is not responding to simple medications? The wrong answer is to give more and more medications. This is underscored by three key HIC papers:

1. The BADGER study compared the effects of either adding LABA, or LTRA, or more than doubling the dose of ICS in children symptomatic on fluticasone 100 mcg bd. Very few children showed any benefit from the increased ICS dosage [10].
2. A study to determine which of adding azithromycin or LTRA to the regimen of children symptomatic on LABA and ICS ended in futility because most children screened for “severe asthma” were either not taking their treatment or did not have asthma [11].
3. An inner city asthma study attempted to determine whether measuring FeNO improved asthma control over and above standard asthma protocols. In the 2-wk run-in period, when protocols were strictly implemented, asthma control improved so much that there was no scope for further improvement with enhanced monitoring [12].

The correct answer is a detailed evaluation of the child to find out what factors are impairing treatment response. This is done through a detailed, multidisciplinary assessment, which most often can resolve the problem [13].

The areas covered in the assessment are as follows:

- Medication adherence: Poor adherence or incorrect use of inhaler devices is the single commonest cause of treatment failure. This cannot be assessed by asking the child and family, who inevitably say they always take treatment regularly. Prescription refills are useful; if the child has not picked up prescriptions they cannot be taking treatment, if they are collecting them, they still may not be using them correctly. Electronic adherence monitors which record activation but not inhalation (newer devices can do both) are used. Directly observed therapy (DOT) may be an option in LMICs.
- Adverse environmental exposures: In HICs, exposure to allergens to which the child is sensitized, especially house dust mite and furry pets is important; asthma attacks are most likely in those sensitized to a high allergen burden home who get a respiratory virus infection [14]. Passive exposure to tobacco and vaping is another all-too-common exposure, and referral of parents to a smoking cessation clinic, if available, should be proposed. Environmental exposures may be particularly challenging in LMICs, where additional exposure to indoor pollution due to biomass fuel and chemicals such as benzene are very common [15].
- Psychological factors: Anxiety and depression are very common in asthma. These are treated in parallel with asthma; it is not useful to try to determine whether anxiety caused asthma, or asthma caused anxiety. Both should be treated on their merits. Psychological stress related to post-traumatic stress disorder and intimate partner violence within the home, and neighborhood poverty and deprivation are all too common in LMICs, and often intractable social problems [15].
- Breathing pattern: Dysfunctional breathing including hyperventilation and exercise-induced laryngeal obstruction (EILO) is common in severe asthma and is often confused with symptoms due to asthma. A respiratory physiotherapist assessment is very helpful. EILO results from vocal cord adduction. It occurs during exercise, unlike exercise-induced bronchoconstriction, which comes on after exercise, leading to a sensation of being unable to breathe in and sometimes stridor. A smartphone video-recording of an attack is often diagnostic, although objective confirmation can be obtained by laryngoscopy during exercise [16]. Breathing re-training by the physiotherapist is often helpful.
- Symptom perception: In some children referred for evaluation, there appears to be a disconnect between reporting of severe symptoms and objective measures of severity. If possible, the view of the school is sought; if the so-called “severe asthmatic” is captain of football team, the diagnosis needs to be reconsidered. Sometimes an admission to hospital is illuminating, with the symptoms closely monitored and exercise being supervised. If there is doubt, an exercise test should be performed; in one study, half of the adolescents complaining of breathlessness had neither exercise-induced asthma or EILO, but were just deconditioned [17].
- Comorbidities are also identified and addressed as far as possible. Any prominent upper airway disease is treated with topical steroids and antihistamines. Any food allergies are confirmed by double blind challenge; food allergy is associated with worse asthma outcomes. Weight-loss advice is given to the obese, but again, this disease of poverty may be intractable. Gastro-oesophageal reflux is frequently identified, but treatment rarely, if ever, makes any difference to asthma outcomes.

The outcome of this assessment is that a very few children will be identified as having STRA and undergo detailed airway phenotyping, preparatory to being considered for biological therapies. For the rest, a bespoke intervention is proposed, addressing the issues identified, which hopefully resolves the problem. If the child and family are unable to follow the plan, this is considered “refractory” asthma and is managed accordingly (below).

## Specific Management Issues: Refractory Asthma Plus

The usual underlying cause is obesity with failed weight loss. In some settings (e.g., severe coexisting metabolic syndrome), bariatric surgery may be contemplated. If this is not an option, the child is reassessed in detail because obese asthma may be phenotypically different from difficult asthma:

- Are the symptoms due to asthma or just deconditioning (above)?: If in doubt, carry out an exercise test; it is a cardinal error to escalate asthma therapies for nonexistent bronchospasm.
- What is the nature of any airway inflammation?: Obesity does not protect against eosinophilic atopic inflammation, but an alternative, non-type 2 inflammatory mechanism which does not respond to ICS is IL6-driven airway inflammation due to a systemic inflammatory state driven by obesity [18].
- What is the nature of airflow obstruction?: Obese asthma patients may have evidence of dysanaptic airway growth (longer than normal airways of normal caliber, a raised total lung capacity with FEV_1_ normal, FVC elevated, and FEV_1_:FVC ratio reduced [19]. Furthermore, variable airflow obstruction may be due to atelectasis secondary to reduced chest wall compliance rather than bronchospasm.

Many of the above are not treatable, but appreciating these possible factors may prevent the obese child being given unnecessary high-dose medications.

## Specific Management Issues: Refractory Difficult Asthma

This usually relates to adherence issues; the child and family being unable or unwilling to take medication regularly. It is of course important to determine what happens if medication is taken regularly; a child with STRA may have stopped taking medications because they are not effective. DOT is used at school, or occasionally remote video monitoring. If there is doubt an admission for DOT will settle the matter. It cannot be overemphasized that poor adherence is a risk for asthma death, and should be taken as seriously as STRA. Indeed, escalation to biologicals (below) is done in such cases, because clinicians have a duty of care to keep the child alive even if the family are unable or unwilling to administer standard medications [20]. Numerous strategies have been employed with varying success to improve adherence, including electronic reminders and even financial incentives, but adherence remains perhaps the most important unsolved problem in pediatric asthma.

## Specific Management Issues: STRA

Most studies of the pathology of STRA are from HIC settings, and clearly show that the disease is heterogeneous. No clear signal emerges, and T helper (TH)-1, TH-2, TH-17 and infective and inflammatory patterns have all been described [21]. In an HIC setting, the next step would be to phenotype the airway using bronchoscopy, bronchoalveolar lavage and endobronchial biopsy. In the author's clinic, phenotypes of either persistent, steroid-resistant airway eosinophilia or a paucigranulocytic picture are seen [22]. There seems little point in applying anti-inflammatory or antieosinophilic strategies to a child who appears to have a noninflammatory phenotype any more than insulin given to a normoglycemic child. The noneosinophilic phenotype is extremely challenging, and trials of long-acting muscarinic agents and azithromycin should be considered. For the eosinophilic patients, in an HIC setting, therapy with a biological (omalizumab or mepolizumab) [23] would be commenced, but the cost of these agents is a challenge in LMICs.

There are additional issues to consider. Omalizumab is indicated in HIC children 6 y and over, if the serum total IgE lies between 76 and 1500, there is evidence of aeroallergen sensitization if the child has had multiple asthma attacks. Mepolizumab is indicated in the same age range if the blood eosinophil count is ≥ 150 μL^−1^, based on adult thresholds. However in LMICs, both a raised IgE and blood eosinophil count may be driven by parasitemia, and there are no LMIC cut-points for these medications. Furthermore, eosinophils may be beneficial, not merely in host defence against parasites, but possibly also against at least some bacterial and viral infections [24], as well as having important roles in normal immunological development [25]. Thus, particularly in a high infection burden setting as in many LMICs, the biologicals could have unexpected harmful effects. Hence if these agents are to be deployed in LMICs, firstly the cost has to come down; and secondly, there needs to be research in LMICs as to the biomarkers and indications for their use. Sadly, the first is unlikely to happen, making the second point largely irrelevant. The treatment of true STRA in LMICs is likely to be substandard for the foreseeable future, largely related to the cupidity of Pharma.

## The Components of Risk in Asthma

### Risk of Medication Side-Effects

Sadly, this is likely to be less of a risk in LMICs because of poor access to medications. If high-dose ICS are needed to control asthma, there is a risk of causing adrenal insufficiency, which may present acutely with hypoglycemia [26], and more chronically with growth failure. In adults, ICS usage is associated with an increased risk of pneumonia, tuberculosis, and atypical mycobacterial infection [27]. This should not deter the appropriate use of ICS, but cautions against indiscriminate prescription.

### Long-Term Respiratory Outcomes

Numerous studies, summarized elsewhere [28], have shown that spirometry tracks from the preschool years at least to late middle age, and early impairment of spirometry, as seen commonly in asthma, is a major risk factor for chronic obstructive pulmonary disease (COPD) in adult life. Lung growth is adversely impacted by postnatal exposure to pollution and the occurrence of asthma attacks in children not using ICS [29]. The challenge in a LMIC setting, given the scarcity of spirometers, is to measure lung function and try to protect especially those with impairment from smoking and other adverse exposures.

### Long-Term Systemic Morbidity and Mortality Including Transgenerational Risk

In HIC settings, low lung function is also a risk factor for premature and increased all-cause morbidity and mortality, including cardiovascular and metabolic [30]. Furthermore, there is a strong correlation between parental spirometry and that of the child, propagating cycles of deprivation. There is no comparable LMIC research, but it seems likely that the relationships will be the same.

## Summary and Conclusions

There remain numerous challenges for those treating asthma in a LMIC setting.

- Asthma differs in its treatable traits across the globe, and even within countries; there are huge pockets of poverty in HICs and affluence in LMICs, and urban–rural differences also vary with farming practice worldwide, meaning that it is imperative to phenotype asthma locally in LMICs, and not rely on HIC data.
- In many LMICs, strong political action to ensure all children have access to the basic necessities of asthma (low-cost ICS and SABA, a milk-bottle spacer, and prednisolone) and enable them to be protected from outdoor and particularly indoor pollution is essential, but often currently lacking.
- In many LMICs, there are barriers such as lack of access to education and stigma based on wrong perceptions which prevent asthma diagnosis and treatment being implemented effectively.
- In many LMICs, there is a need to address both overuse and underuse of ICS, both of which are real issues in both LMIC and HIC settings.
- There is an important role for education modules in schools of medicine and nursing, discussed elsewhere in this issue, and national societies have an important role to play driving up standards and maintaining political pressure to ensure that all children have access to high-quality care.
- There is no research at all in the novel biologicals, especially the indications and the use of predictive biomarkers in LMICs. This is currently logical since these agents are prohibitively expensive. It is a scandal that Pharma has been allowed to get away with ignoring these needs. It does not need to be so; the example of the provision of cheap high-activity antiretroviral treatment (HAART) to subSaharan Africa shows what can be done if there is a will to do it.

There are many ways in which asthma treatment in LMICs can be improved. The author's challenge to young investigators reading this review is to go out and drive that change!
